# Genetic association of the transcription of neuroplasticity-related genes and variation in stress-coping style

**DOI:** 10.1002/brb3.360

**Published:** 2015-06-24

**Authors:** Saeko Aizawa, Yoshinobu Ishitobi, Koji Masuda, Ayako Inoue, Harumi Oshita, Hirofumi Hirakawa, Taiga Ninomiya, Yoshihiro Maruyama, Yoshihiro Tanaka, Kana Okamoto, Chiwa Kawashima, Mari Nakanishi, Haruka Higuma, Masayuki Kanehisa, Jotaro Akiyoshi

**Affiliations:** 1 Department of Neuropsychiatry, Oita University Faculty of MedicineHasama-Machi, Yufu-Shi, Oita, 879-5593, Japan; 2 Department of Applied Linguistics, Oita University Faculty of MedicineHasama-Machi, Yufu-Shi, Oita, 879-5593, Japan

**Keywords:** BDNF, ego aptitude scale, Lazarus-type stress-coping inventory, NTRK2, polymorphism, social adaptation self-evaluation scale

## Abstract

**Introduction:**

Stress coping has been defined as the cognitive and behavioral efforts made to conquer, endure, or decrease external and internal demands and the conflicts between them. It has two main elements: the control or modification of the person–environment relationship causing the stress (i.e., problem-focused coping) and/or regulation of stressful feelings (i.e., emotion-focused coping). Research suggests that the expressions of brain-derived neurotrophic factor (BDNF) and neurotrophic tyrosine kinase receptor type 2 (NTRK2) play important roles in brain adaptation to investigate stress. To clarify the genetic basis of stress coping, we investigated the association of stress-coping strategies and social adaptation with single-nucleotide polymorphisms (SNPs) involved in neural plasticity, anxiety, and depression.

**Methods:**

In 252 healthy controls (94 women; 158 men), we measured and estimated the stress-coping style using the Lazarus-type stress-coping inventory, ego aptitude scale (EAS), and social adaptation self-evaluation scale (SASS). We investigated one SNP of BDNF (rs6265, Val/Met) and five SNPs of NTRK2 (rs11140800, rs1187286, rs1867283, rs1147198, and rs10868235).

**Results:**

We observed significant associations between BDNF and emotion-focused strategies, seeking social support, self-control, and distancing. We also found significant associations between NTRK2 and cognitive strategies, problem-solving, confrontive- coping, seeking social support, distancing and positive reappraisal. Significant associations were also found between BDNF and critical attitudes and between NTRK2 and all seven ego-related factors on the EAS. In the SASS, the minor allele rs1867283 of NTRK2 had a significantly higher score than the heterozygote.

**Conclusions:**

These findings may provide insights into the partial effects of genetic mutations in BDNF and NTRK2 on stress tolerance and personality.

## Introduction

Stress coping is defined as the cognitive and behavioral efforts made to conquer, endure, or decrease external and internal demands and the conflicts between them (Folkman et al. 1986). Lazarus and Folkman (1984a) have defined stress coping as “actual strategies used to mediate primary and secondary appraisals”. Coping styles involve two primary coping strategies: problem-focused coping, which seeks to control or modify the relationship between the person and the actors causing stress, and emotion-focused coping, which seeks to regulate stressful feelings. In addition, Lazarus and Folkman (1984a,b) proposed eight strategies that are used for relieving stress: planful problem-solving (Pla), confrontive coping (Con), seeking social support (See), accepting responsibility (Acc), self-controlling (Sel), escape-avoidance (Esc), distancing (Dis), and positive reappraisal (Pos). All these strategies are important for relieving stress and preventing depression, but different individuals may prefer different coping strategies. The traits or differences leading an individual to prefer a particular coping strategy are unclear. Indeed, specific genetic factors may play a key role. Here, we therefore focused on the roles of brain-derived neurotrophic factor (BDNF) and neurotrophic tyrosine kinase receptor type 2 (NTRK2), which have been shown to play important roles in brain adaptation to stress.

To examine how people deal with stress, it is necessary to consider the factors that have a significant impact on their life style. Stress coping tends not to change relative to stressful situations, which is partially related to a person’s personality. The Ego Aptitude Scale (EAS), created based on “Adaptation to Life” by Vaillant (1977) and “Born to Win” by James and Jongeward (1971), can help in this setting. It is presented as questionnaire with an emphasis on motivational elements of personality variables when an individual acts. Lazarus argued that stress begins with the perceptions of a threat to one’s self; thus, health is maintained when self-evaluation by the person to cope with the stressor is high, and the disease or stressor is enhanced when self-assessment is low (Lazarus and Folkman 1984a,b). To have this confidence and high self-esteem enables a person to address situations and interpersonal relationships effectively and to have a sense of fulfillment in life. Lazarus described that it was difficult to identify the patterns of stress coping; however, it was Lazarus assumed that this problem would be solved because the social relationships and personality traits of a person affect the stress pattern. The EAS uses measures that meet the conditions of the day-to-day stress theory of Lazarus, motivation at the time, and stress management techniques, and should be effective in the estimation of personality characteristics related to stress. Therefore, this scale should fit with the overall objectives of examining the relationship between neuroplasticity-related genes and stress coping.

Lazarus and Folkman (1984a) defined the stress coping as “Constantly changing cognitive and behavioral alternatives to manage specific external and /or internal demands that are appraised as taxing or exceeding the resources of the person”. Such coping styles provide two primary coping strategies: emotional regulation, which involves strategies aimed at changing the way one thinks or feels about a stressful situation and problem management, which strategies directed at changing a stressful situation (Lazarus and Folkman 1984b). In addition, Lazarus et al. developed eight coping methods that relieve stress: Pla, Con, See, Acc, Sel, Esc, Dis, and Pos (Folkman and Lazarus 1988).

Chronic stress induces hippocampal atrophy and reduces the expression of BDNF in limbic structures, including the hippocampus and prefrontal cortex, involved in the regulation of mood and cognition (Nibuya et al. 1995; Smith et al. 1995). BDNF overexpression prevents chronic stress-induced anxiety and has an antidepressant effect (Govindarajan et al. 2006). Recently, BDNF has been associated with acute and chronic stress-induced structural plasticity in both the hippocampus and amygdala (Lakshminarasimhan and Chattarji 2012). The release of BDNF in the hypothalamus is also associated with adaptive changes during the stress response (Givalois et al. 2004). In a recent study, coping with mild intermittent stress-induced adult neurogenesis in the hippocampal dentate gyrus (Parihar et al. 2011).

Resilience, the ability to cope with stressful situations and develop adequate behavioral and psychological adaptation to chronic stress (McEwen 2007; Feder et al. 2009), may also be related to BDNF. Persistent alterations in BDNF after social stress allow neural adaptation in the amygdala and ventral tegmental area (Fanous et al. 2010). Life stresses such as childhood adversity or a recent stressful event, are recognized as being risk factors for depression (Hosang et al. 2014). Notably, the Met allele of BDNF, Val66Met, has been shown to have significant associations with life stress and depression.

The tyrosine kinase B (TRKB) receptor, which is stimulated in the hippocampus and prefrontal and anterior cingulate cortex antidepressant treatment, is vital for producing antidepressant effects (Saarelainen et al. 2003). NTRK2 is a specific TRKB receptor for BDNF and has a regulatory role in neural differentiation and in the maintenance of specific neuron populations in areas such as the human prefrontal cortex (Luberg et al. 2010). Five of its genotyped polymorphisms are located on chromosome 9. BDNF/NTRK2-stimulated intracellular signaling considerably affects the actions of antidepressants (Duman and Monteggia 2006). Thus, several studies have examined the relationship between depression and BDNF and NTRK2, although few have examined how these genes contribute to an individual’s vulnerability to stress. We hypothesized that BDNF and NTRK2 influence stress-coping styles.

BDNF and NTRK2 reportedly play important roles in the adaptation of the brain to stress (Cowansage et al. 2010; Autry and Monteggia 2012; Lu et al. 2013; Wang et al. 2013). BDNF polymorphisms involve the substitution of valine (Val) with methionine (Met), resulting in three variants (Val/Val, Val/Met and Met/Met). The BDNF Met allele causes inefficient BDNF secretion (Egan et al. 2003) and decreases in BDNF leads to vulnerability to disorders such as depression and anxiety (Karege et al. 2002; Martinowich et al. 2007). Individuals who have experienced childhood adversity and who carry BDNF variants (rs6265) have an increased risk for lifetime depression (Juhasz et al. 2011; Perea et al. 2012). Similar to BDNF, NTRK2 also demonstrates antidepressant-like effects (Saarelainen et al. 2003; Blugeot et al. 2011). Although several studies have examined the relationships between depression and BDNF or NTRK2, few have examined whether these genes contribute to an individual’s stress-coping style.

Recent research into stress has focused on the role of resilience (Feder et al. 2009). The development of pathological stress does not necessarily depend on the severity of the stress, as evidenced by the fact that many people who have experienced trauma do not develop psychopathology (Charney 2004). Indeed, the brain demonstrates a major plasticity under stress loads, with genomic and nongenomic changes and the reformation of neural connectivity. Some of these events are associated with an increase in risk that leads to pathological consequences, whereas others are involved in the development of resilience (Vialou et al. 2010). Stress resilience therefore appears to be a neural adaptation to stress that is probably facilitated by a person’s genetic constitution.

Different individuals may utilize different coping styles but the traits that lead individuals to prefer one coping style to another are unclear. We hypothesized that genes involved in neuroplasticity are associated with these differences in stress-coping styles. Therefore, this study aimed to investigate the association of single-nucleotide polymorphisms (SNP) involved in neuroplasticity with stress-coping strategies, ego aptitude, and social adaptation.

## Materials and Methods

### Participants

We enrolled an ethnically homogeneous Japanese sample of medical students from Oita University with no history of any mental disorders or psych pharmacotherapy. We excluded participants who took any medication during the tests, as well as those with any mental disorders identified by the Mini-International Neuropsychiatric Interview (MINI) and DSM IV Axis II Disorders (SCID-II) questionnaire.

In total, 252 healthy participants were recruited, of which 158 were men and 94 were women, with an age of 24.5 ± 2.7 years (mean ± SD). The participants were asked to complete the Lazarus-type Stress-Coping Inventory (SCI), EAS, and Social Adaptation Self-Evaluation Scale (SASS). Six men did not answer the SASS questions; thus, 152 men and 94 women (total, 246) participated in that test. All participants volunteered for this study and gave written informed consent. The study was approved by the Ethics Committee of Oita University.

### Assessment of stress-coping style and anxiety

#### The SCI

The Ways of Coping Questionnaire (WCQ), which Folkma and Lazarus (1988) used to assess coping strategies, was translated by the Japanese Association of Health Psychology into Japanese and became the SCI (Japanese Institute of Health Psychology 1996). This assesses emotion- and problem-focused strategies with eight coping methods: Pla, Con, See, Acc, Sel, Esc, Dis, and Pos. The highest score for each strategy was 60 and that for each method was 16.

#### The EAS

This tool was created by the Japanese Association of Health Psychology based on transactional analysis. Berne (1961) defines alternating analyses as a method to study the interaction between people. Transactional analysis has been developed to understand the process of communication and the ego state of mind during conversation with others. EAS assesses seven ego aptitudes (critical, nurturing, mature, rational, natural, intuitive, and adaptive), with the highest score per attitude being 12.

#### The SASS

Devised by Bosc et al. (1997), this consisted of 21 items for evaluating social motivation and awareness factors that affect the degree of improvement of depression (Goto et al. 2005). The highest score is 60 (the first two questions are “either/or”). A person who achieved a higher score had a better ability to adapt socially; the reliability and validity of the Japanese version of SASS were subsequently confirmed (Bosc et al. 1997).

### DNA extraction and genotyping

DNA was extracted from leukocytes using the DNA Extractor SP Kit (WAKO, Osaka, Japan). After precipitation with ethanol, the DNA pellet was suspended in 50 *μ*L of the specific buffer provided with the kit, quantified by spectrophotometry, and stored at −80°C. We investigated one BDNF SNP (rs6265, Val/Met) and six NTRK2 SNPs (rs1147198, C/A; rs1187286, C/A; rs1867283, G/A; rs10868235, C/T; and rs11140800, C/A). These were genotyped by polymerase chain reaction (PCR) using the Taqman® PCR SNP genotyping assay and a Light Cycler480 (Roche, Basel, Switzerland). The standard 10-*μ*L PCR reaction, which included 2 ng of genomic DNA, was performed using the Taqman® PCR with Universal PCR Master Mix under the protocol guidelines.

### Statistical analysis

A one-way analysis of variance (ANOVA) was used to determine significant differences among genotypes (major and minor alleles). A multivariate ANOVA was used to determine significant differences in the mean scores of the SCI, EAS, and SASS. The extent of linkage disequilibrium between the polymorphisms was calculated and presented graphically with Haploview ver. 4.1. (Barrett et al. 2015). When the difference was significant (*P *<* *0.05), multiple comparisons by the Holm’s step-down method were used. Then, the scores of all groups were evaluated to determine the significant high or low score based on a significance level of *P *<* *0.05. Bonferroni correction was used to adjust for multiple testing. Statistical analysis of single or multiple SNPs were conducted using SNP Stats (Solé et al. 2006).

## Results

**Figure 1:**
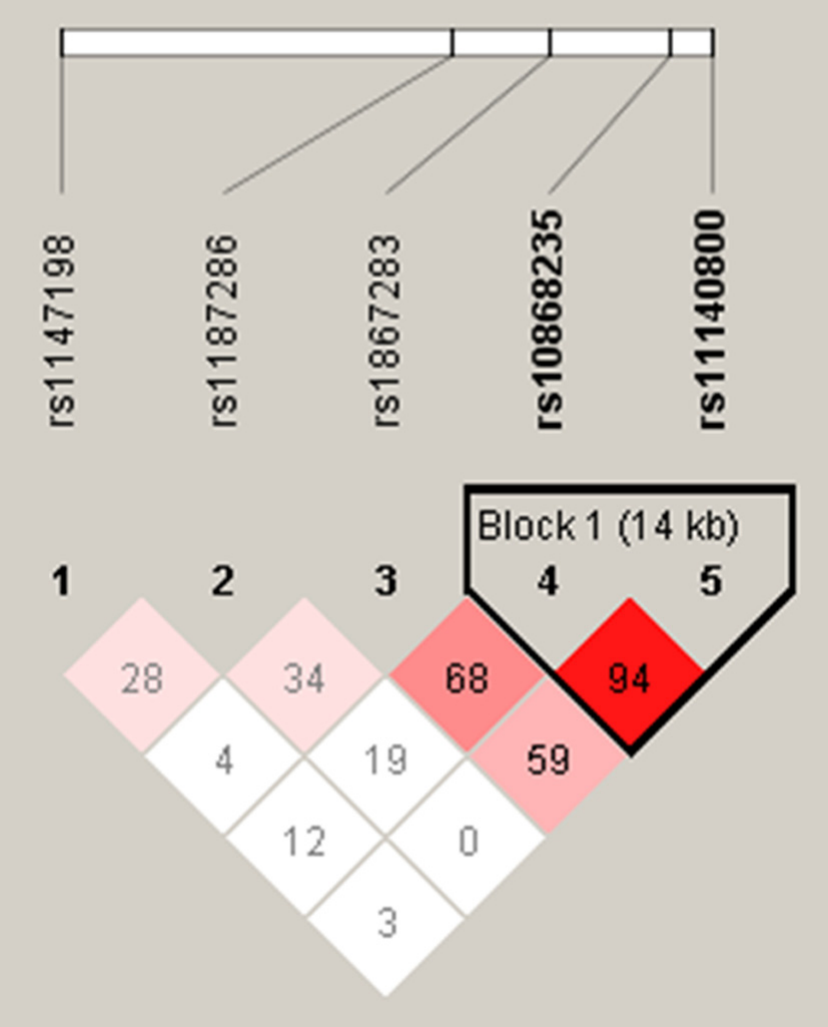
Linkage disequilibrium (LD) plot of the neurotrophic tyrosine kinase receptor type 2 (NTRK2) gene. The LD plot was generated by Haploview software using genotype data from this study. The pairwise *r*^2^ level, which indicates the correlation between two single-nucleotide polymorphisms (SNPs), is shown in gray scale with its values described as the percentage in each cell. Two SNPs within the NTRK2 gene formed one block.

For each of the 252 participants, genotype distributions were determined to be in Hardy- Weinberg equilibrium for all SNPs (Table 2005). Using SNP genotyping data in this study, only one LD block was constructed by the Gabriel algorithm (Gabriel et al. 2002). The block consisted of two SNPs (rs10868235 and rs11140800) within the promoter region of the NTRK2 gene (Fig.1). Two common haplotypes (frequencies ≥0.05 for each) with a cumulative frequency of 99.89% were identified.

**Table 1:** Genotype of the SNPs in the BDNF and five NTRK2 and STAI and BDI

BDNF		rs6265				*P* for HWE
		Val/Val	Val/Met	Met/Met		
	*n*	86	115	51		0.27
STAI	Trait	42.4 ± 9.7	40.3 ± 9.9	41.2 ± 9.0	GG > GA*	
	State	39.0 ± 9.5	37.1 ± 8.9	38.1 ± 9.3		
BDI		3.0 ± 4.1	4.1 ± 5.6	3.3 ± 4.7		

NTRK2		rs11140800				
		CC	CA	AA		
	*n*	10	78	164		0.85
STAI	Trait	43.3 ± 11.9	40.9 ± 9.6	41.3 ± 9.5		
	State	37.0 ± 12.8	37.8 ± 8.8	38.3 ± 9.2		
BDI		8.0 ± 11.1	2.7 ± 3.1	3.6 ± 5.0	CC > CA**, AA*	

NTRK2		rs1187286				
		CC	CA	AA		
	*n*	18	109	125		0.38
STAI	Trait	41.7 ± 10.0	40.7 ± 10.2	41.6 ± 9.0		
	State	38.5 ± 9.8	37.2 ± 8.8	38.8 ± 9.6		
BDI		6.9 ± 8.0	2.6 ± 4.0	3.9 ± 4.3	CC > CA**, AA*	

NTRK2		rs1867283				
		GG	GA	AA		
	*n*	35	111	106		0.49
STAI	Trait	42.4 ± 9.7	40.9 ± 9.4	41.0 ± 9.8		
	State	39.2 ± 10.9	37.6 ± 8.7	38.1 ± 9.1		
BDI		5.5 ± 7.4	3.1 ± 3.5	3.3 ± 5.2	CC > CA*, AA*	

NTRK2		rs1147198				
		CC	CA	AA		
	*n*	32	109	111		0.52
STAI	Trait	40.1 ± 10.1	42.0 ± 9.7	41.0 ± 9.5		
	State	37.6 ± 10.4	37.9 ± 8.5	38.4 ± 9.6		
BDI		3.5 ± 5.6	3.6 ± 5.2	3.5 ± 4.5		

NTRK2		rs1086823				
		CC	CT	TT		
	*n*	163	77	12		0.46
STAI	Trait	41.6 ± 9.7	40.4 ± 9.4	43.2 ± 11.5		
	State	38.7 ± 9.3	37.2 ± 8.8	38.1 ± 12.0		
BDI		3.5 ± 4.9	3.0 ± 3.3	6.8 ± 10.4	CT < TT*	

BDNF, brain-derived neurotrophic factor; STAI, State-Trait Anxiety Inventory; BDI, Beck Depression Inventory; HWE, Hardy–Weinberg equilibrium; SNP, single-nucleotide polymorphisms; NTRK2, neurotrophic tyrosine kinase receptor type 2.

* *P* < 0.05

** *P* < 0.01.

### BDNF

Em, See, Sel, and Dis in the SCI, and critical attitude in EAS significantly differed among rs6265 BDNF genotypes but there were no significant differences observed in SASS.

In the SCI, the major Met/Met allele was associated with significantly higher scores than other genotypes in the Em (*F* = 8.09, *P* < 0.01, df = 2) and See (*F* = 7.12, *P* < 0.01, df = 2) domains. For Sel and Dis in the SCI, Met/Met was associated with significantly higher scores than the Val/Met heterozygote; however, there were no significant differences between the Val/Val allele and the minor Val/Met allele (*F* = 5.18, *P* < 0.01, df = 2 and *F* = 6.87, *P* < 0.01, df = 2, respectively; Table 2002). In the EAS, Met/Met was associated with a significantly lower score than the other genotypes in the critical attitudes domain (*F* = 7.11, *P* < 0.01, df = 2; Table 2002). There were no significant differences in SASS scores between the major and minor alleles (*F* = 1.14, *P* = 0.32, df = 2).

**Table 2:** Comparison of SCI, EAS, and SASS to the genotypes of BDNF and NTRK2 (*P*-value)

Gene	BDNF	NTRK2				
	rs6265	rs11140800	rs1187286	rs1867283	rs1147198	rs10868235
SCI						
Cognitive strategies	0.109	**<0.05**	**<0.05**	0.988	0.330	**<0.05**
Emotion focused strategies	**<0.001**	0.450	0.260	0.333	0.696	0.374
Planned problem-solving	0.398	0.071	**<0.01**	0.507	0.826	0.178
Confrontative coping	0.586	**<0.01**	0.094	0.349	0.753	**<0.01**
Seeking social support	**<0.001**	**<0.05**	0.264	0.183	0.590	0.453
Accepting responsibility	0.753	0.417	0.202	0.958	0.837	0.145
Self-controlling	**<0.01**	0.267	0.771	0.537	0.264	0.204
Escape-avoidance	0.351	0.806	0.833	0.746	0.290	0.236
Distancing	**<0.0.01**	0.875	**<0.05**	0.757	0.504	0.822
Positive reappraisal	0.665	0.107	0.144	**<0.05**	0.340	0.203
EAS						
Critical Attitude	**<0.001**	0.483	**<0.05**	0.824	0.238	0.936
Nurturing Attitude	0.132	0.271	**<0.05**	**<0.05**	0.576	0.442
Mature Attitude	0.620	**<0.001**	0.229	0.114	0.857	**<0.05**
Rational Attitude	0.345	**<0.05**	0.333	**<0.01**	0.106	0.104
Natural Attitude	0.324	**<0.0001**	0.694	0.233	0.359	**<0.001**
Intuitive Attitude	0.147	**<0.001**	0.258	0.852	0.207	**<0.05**
Adaptive Attitude	0.112	0.636	0.180	<0.05	0.674	0.790
SASS	0.367	**<0.05**	**<0.05**	**<0.05**	0.454	**<0.05**

BDNF, brain-derived neurotrophic factor; NTRK2, neurotrophic tyrosine kinase receptor type 2; SCI, stress-coping inventory; EAS, ego aptitude scale; SASS, social adaptation self-evaluation scale. Bold values are significant.

### NTRK2

Five of the genotyped polymorphisms were located on chromosome 9. Linkage analysis revealed one haplotype block (Table 2002), which comprised the rs1147198 and rs10868235 polymorphisms.

At rs11140800 in NTRK2, we found significant differences between genotypes associated with the Co (*F* = 3.52, *P* < 0.01, df = 2), Con (*F* = 5.33, *P* < 0.01, df = 2), and See (*F* = 3.39, *P* < 0.01, df = 2) in the SCI, and mature attitude (*F* = 8.08, *P* < 0.01, df = 2), rational attitude (*F* = 4.18, *P* < 0.05, df = 2), natural attitude (*F* = 10.44, *P* < 0.01, df = 2), and intuitive attitude (*F* = 7.47, *P* < 0.01, df = 2) in the EAS. In the SCI, the minor A/A allele was associated with a significantly lower score in the Con domain than the other genotypes. However, there were no significant differences among the genotypes in the association with the Co and See domains. In the EAS, the heterozygote A/C allele was associated with significantly higher scores than the other genotypes for mature attitudes, natural attitudes, and intuitive attitudes; the major CC allele was also associated with significantly higher scores than the A/A allele in those attitudes. However, there were no significant differences in the associations of genotypes with the rational attitudes domain (Table 2002).

For NTRK2 rs1187286, we found significant differences between genotypes in the association with Co, Pla, and Dis in the SCI, and with critical attitude and nurturing attitude in the EAS. There were also significant differences in the SASS between genotypes. In the SCI, the minor C/C allele was associated with a significantly higher score than the other genotypes in the Dis domain (*F* = 4.09, *P *<* *0.01, df = 2). For the Co and Pla domains of the SCI, the A/C heterozygote was associated with significantly higher scores than the major A/A allele but there were no significant differences between the A/A and C/C alleles or the A/C and C/C alleles (*F* = 3.66, *P* < 0.01, df = 2 and *F* = 5.69, *P *<* *0.01, df = 2, respectively). In the EAS, the C/C allele was associated with a significantly higher score than the other genotypes in the critical attitudes domain (*F* = 4.41, *P* < 0.05, df = 2). For nurturing attitudes, A/C had a significantly higher score than A/A but there were no significant differences between A/A and C/C or between A/C and C/C (*F* = 4.45, *P *<* *0.05, df = 2; Table 2002).

For NTRK2 rs1867283, significant differences were observed between genotypes for the Pos domain of the SCI and the critical attitude and nurturing attitude domains of the EAS; there were also significant differences in the SASS. In the SCI, the minor A/A allele was associated with a significantly higher score than the other genotypes in the Pos domain (*F* = 3.52, *P* < 0.05, df = 2). On the other hand, in the EAS, it was associated with a significantly higher score than the other genotypes for the rational attitudes domain (*F* = 4.95, *P *<* *0.01, df = 2). The heterozygous A/G allele was associated with a significantly lower score in the adaptive attitudes domain (*F* = 4.57, *P *<* *0.05, df = 2). When nurturing attitudes were examined, the major G/G allele was associated with a significantly higher score than the A/G allele but there were no significant differences between either the G/G and A/A or between the A/G and A/A alleles (*F* = 3.77, *P* < 0.05, df = 2; Table 2002).

For NTRK2 rs1147198, no alleles (G/G, G/T, and T/T) showed significant differences between tests. However, for NTRK2 rs10868235, significant differences among genotypes existed for Co and Con in the SCI, and for mature attitude, natural attitude, and intuitive attitude in the EAS; significant differences also existed in the SASS. In the SCI, the minor T/T allele was associated with a significantly lower score than the other genotypes i in the Con domain (*F* = 6.89, *P* < 0.01, df = 2) but there were no significant differences among the genotypes in the Co domain (*F* = 3.12, *P* < 0.05, df = 2). In the EAS, the heterozygous C/T allele was associated with a significantly higher score than the other genotypes for the natural attitudes domain (*F* = 7.42, *P* < 0.01, df = 2). In addition, the major C/C allele was associated with a significantly higher score than the T/T allele in those attitudes and the C/T allele was associated with a significantly higher score for intuitive attitudes compared with the C/C allele; however, there were no significant differences between either the C/C and T/T or the C/T and T/T alleles (*F* = 3.92, *P *<* *0.05, df = 2). The minor allele in rs1867283 was associated with a significantly higher score in the SASS than the heterozygote (*F* = 3.21, *P *<* *0.01, df = 2) but there were no significant differences between the major and minor alleles or between the heterozygote and minor allele for rs11140800 (*F* = 2.27, *P *=* *0.11, df = 2), rs1187286 (*F* = 2.93, *P *=* *0.06, df = 2), rs1147198 (*F* = 1.88, *P *=* *0.15, df = 2), or rs10868235 (*F* = 1.77, *P *=* *0.17, df = 2; Table 2002).

## Discussion

For BDNF rs6265, the Em, See, Sel, and Dis domains of the SCI, and the critical attitude domain of the EAS significantly differed among the genotypes, whereas there were no significant differences for the SASS results between genotypes. High levels of stress inhibit the production of proteins and BDNF mRNA in the hippocampal area CA1, the dentate gyrus, and hypothalamus (Schaaf et al. 1998, 2000; Aliaga et al. 2002). Although the hippocampus is the center of learning and memory, it is very sensitive and vulnerable to injury (Eichenbaum 1997).

Chronic stress affects dendritic morphology, cognitive function, and neural development and can alter the hippocampal plasticity (McEwen and Sapolsky 1995; Kim et al. 1996; Gould and Tanapat 1999). For example, Met/Met mice have shown decreased hippocampal volume and learning difficulties (Chen et al. 2006) and are comparatively less affected in the social defeat paradigm (Krishnan et al. 2007; Surtees et al. 2006). Conversely, humans with the Met allele (rs6265) have been shown to have hippocampal dysfunction and hypersensitivity to stress (Murakami et al. 2005), suggesting somewhat contradictory findings. In addition, having a “fighting spirit” is associated with a better coping style (Classen et al. 1996), whereas a helplessness/hopelessness stress-coping style is associated with depression in patients with breast cancer (Schou et al. 2004).

Our data showed that individuals with the Met allele of rs6265 scored higher in the Em, See, Sel, and Dis domains of the SCI compared with those with the Val allele. Because individuals with the Met allele display more signs of anxiety, they may have an enhanced stress-coping style with increases in the Em, See, Sel, and Dis domains of the SCI. Our data also showed that individuals with the Met allele had lower scores in the critical attitude domain of the EAS than those with the Val allele. According to the theory of Lazarus and Folkman (1984a), stress coping serves two main functions: the adjustment of distress or emotion and the handling of the problem that caused the distress. Because individuals with the Met allele display more anxiety, they may have a weakened ego attitude. It is important for individuals to have insight into their ego and the effect of relationships with others or with their family, thereby providing objective information about their relationship with stress. Individuals with higher critical attitude scores tend to have more physical and mental health issues (Ashitomi 2005).

In this study, for NTRK2 rs11140800, significant differences existed among genotypes for the Co, Con, and See domains of the SCI and for the mature attitude, rational attitude, natural attitude, and intuitive attitude domains of the EAS. For NTRK2 rs1187286, significant differences existed among genotypes for the Co, Pla, and Dis domains of the SCI and for the critical attitude and nurturing attitude domains of the EAS. For NTRK2 rs1867283, significant differences existed among genotypes for the Pos domain of the SCI and for the critical attitude and nurturing attitude domains of the EAS. For NTRK2 rs10868235, significant differences existed among genotypes for the Co and Con domains of the SCI and for the mature attitude, natural attitude, and intuitive attitude domains of the EAS, with significant differences present in the SASS.

In the rat hippocampus, stress is associated with a compensatory organized decrease in BDNF expression through an increase in the mRNA of TRKB (Nibuya et al. 1999). TRKB-knockout mice display unsuitable coping behaviors under stressful situations (Minichiello et al. 1999; Adams et al. 2005), whereas transgenic mice overexpressing TRKB receptors have increased contextual fear conditioning (Koponen et al. 2004). TRKB protein levels in the hippocampus are higher in rats exposed to a single prolonged stress than in control rats after fear conditioning (Takei et al. 2011). TRKB in the adrenal medulla can also induce catecholamine release in rats subject to 60-min stress situations (Kondo et al. 2013). Thus, the adrenal medulla may provide a positive feedback loop through autocrine BDNF–TRKB interactions under acute stress conditions. Indeed, treatment with the selective norepinephrine reuptake inhibitor, reboxetine, reverses chronic mild stress-reduced hippocampal BDNF levels and stress-increased TRKB levels (First et al. 2013). However, chronic and acute stresses do not change NTRK2 expression in the hippocampus (Vellucci et al. 2001).

In our study, there was no association between any of the tested SNPs for either NTRK2 or BDNF and major depressive disorder (Kohli et al. 2010). In addition, the minor allele of NTRK2 rs1867283 was associated with a significantly higher SASS score than the heterozygote. The SASS is a relatively recent test that was developed to measure aspects of motivation-related social function (Bosc et al. 1997). Notably, a significant positive relationship has been observed between the SASS and interpersonal relationship factor scores and prefrontal cortex activation and between SASS interest and motivation factor scores and prefrontal cortex activation (Pu et al. 2014).

This study has some limitations. First, we measured stress-coping styles by the SCI, EAS, and SASS. These inventories are self-administered tests, so further objective testing would be necessary to support our results. Second, we used six SNPs, one of BDNF (rs6265) and five of NTRK2 (rs11140800, rs1187286, rs1867283, rs1147198, and rs10868235). However, because it is impossible to exclude the possibility that other SNPs are associated with stress, the number of studied SNPs for BDNF and NTRK2 should be increased. Third, we used young participants in this study. Because differences in age may induce differences in stress-coping styles, the age range of the participants should also be widened to assess the effect of this variable. Fourth, our sample size was insufficient given the substantial number of tests conducted, which was particularly relevant given the infrequency of some of the minor alleles of the polymorphisms. However, this was somewhat compensated by the use of correction for multiple comparisons. Further studies are needed with larger sample sizes. Finally, this study had limited power and it was notable for lacking haplotype testing.

In conclusion, our results indicate that common, functionally significant polymorphisms in BDNF and NTRK2 partially modulate stress-coping strategies, depression, and anxiety. In addition, seven ego-related factors were associated with the presence of BDNF and NTRK2 polymorphisms. It is possible that social adaptation is associated with the stress-coping style and ego attitude adopted by an individual.
